# Prognostic and Clinical Implications of UNC13C expression in Hepatocellular Carcinoma Patients

**DOI:** 10.7150/ijms.80488

**Published:** 2023-08-06

**Authors:** V. Bharath Kumar, Chien-Hsun Lee, Tzu-Cheng Su, Chia-Chieh Lin, Zuhair M. Mohammedsaleh, Chung-Min Yeh, Rudolf Kiefer, Shu-Hui Lin

**Affiliations:** 1 Department of Medical Laboratory Science and Biotechnology, Asia University, Taichung, Taiwan.; 2 Department of Pathology, E- Da Hospital, I-Shou University, Kaohsiung, Taiwan.; 3 Department of Surgical Pathology, Changhua Christian Hospital, Changhua, Taiwan.; 4 School of Medicine, Chung Shan Medical University, Taichung 402, Taiwan.; 5 Oral Cancer Research Center, Changhua Christian Hospital, Changhua, Taiwan.; 6 Department of Medical Laboratory Technology, Faculty of Applies Medical Sciences, University of Tabuk, Tabuk-71491, Kingdom of Saudi Arabia.; 7 Conducting Polymers in Composites and Applications Research Group, Faculty of Applied Sciences, Ton Duc Thang University, Ho Chi Minh City 758307, Vietnam.; 8 Department of Medical Laboratory Science and Biotechnology, Central Taiwan University of Science and Technology, Taichung, Taiwan.; 9 Department of Post-Baccalaureate Medicine, College of Medicine, National Chung Hsing University. Taichung, Taiwan.

**Keywords:** UNC13C, HCC, prognosis, T stage, survival, independent prognostic

## Abstract

Aberrant expression of UNC13C (Unc-13 Homolog C) has been observed during the progression of oral squamous cell carcinoma. However, the expression pattern and clinical relevance of UNC13C in Hepatocellular carcinoma (HCC) remain to be elucidated. The purpose of this study is to examine UNC13C expression in HCC and explore its role in clinicopathological factor or prognosis in HCC. Two hundred and sixty-five patients diagnosed with HCC were included in the present study. The expression of UNC13C in HCC tissues was analyzed by immunohistochemistry analysis. The relationship between UNC13C protein and clinicopathological characteristics in HCC was investigated. Moreover, the high expression of UNC13C was significantly correlated with T stage, AJCC stage and overall survival rates. Cox regression analysis identified UNC13C as an independent prognostic indicator for HCC patients. UNC13C might be a prognostic biomarker and therapeutic target in HCC. Further studies with larger sample sets are needed to understand the clinical implications of UNC13C in hepatocellular carcinoma.

## Introduction

Recent epidemiological studies showed that Hepatocellular carcinoma (HCC) accounts for the third major cause of cancer-related deaths 1. Globally HCC incidence is increasing, with an estimated range of 600,000-800,000 new cases occurring yearly 2-4. Multiple factors are involved in the etiology of HCC 5 and the risk factors includes, Hepatitis B virus (HBV) 6 and, hepatitis C virus (HCV) infection 7, 8, alcoholic fatty liver disease (AFLD), and non-alcoholic fatty liver disease (NAFLD). The prognosis of HCC remains poor due to ideal diagnosis and the treatment in HCC 9. Therefore to improve the survival rate of HCC patients, it is of essential to identify new targets for early diagnosis and treatment.

Unc-13 homolog C (UNC13C), a member of the Unc/Munc family is involved in aspects of cancer progression such as tumour development, cell migration 10, and metastasis. Furthermore, low expression of UNC13C has been suggested to predict poor prognosis of oral cancer in a study examining the expression of OSCC in 268 patients 10. The anti-cancer role of UNC13C in OSCC was confirmed by Dong, et al 2021^11^. Apart from this, UNC13C plays a major role in vesicle maturation during exocytosis 12, and in neurotransmitter release 13. However, there is a great need to further elucidate the function of UNC13C protein expression in HCC should also be explored.

In the current study, immunohistochemical (IHC) analysis was used to examine the expression of UNC13C in HCC patients. We also examined the relationship between UNC13C protein expression and HCC clinicopathological variables and prognosis. Finally, we wanted to identify potential prognostic biomarker to help in early detection of hepatocellular carcinoma (HCC).

## Materials and Method

### Participants and Clinical tissues

Hepatocellular carcinoma patient's tissues (n=265) were obtained from the Department of Pathology at Changhua Christian Hospital, Taiwan. This research was approved by the Ethics Committee of Changhua Christian Hospital (CCH IRB No. 190603) to use decoded tissue samples. IRB agreed to use formalin-fixed, paraffin-embedded decoded tissue array samples without informed consent. Patient information has been decoded and identifiable data were removed. IRB approved to use formalin-fixed, paraffin-embedded (FFPE) decoding tissue microarray samples without informed consent.

### Immunohistochemistry

Immunohistochemistry analysis was performed according to the standard protocol as previously described 14. Two pathologists independently evaluated and scored the stained slides. The staining intensities were scored no staining, negative, low/weak positive, 1+; strong positive, 2+). The American Joint Committee on Cancer (AJCC, 7th Edition) Tumor, Node, Metastasis (TNM) staging system and the Edmondson-Steiner grading system were used to make pathological evaluations of tumor stages and histological differentiation. The primary antibodies involved in the immunohistochemistry analysis: UNC13C antibody (1:40; ab122725, Abcam, Cambridge, UK).

### Statistical analysis

All analyses were performed using Statistical Product and Service Solutions (SPSS, version 17) (SPSS, Inc., Chicago, IL, USA). Fisher's exact test or the Chi-square test was used to detect the importance of the clinicopathological variables of cytoplasmic UNC13C protein expression and HCC. The overall survival (OS) time, was estimated with the Kaplan-Meier method and compared using the Log rank test. Univariate and multivariate analysis was performed to confirm prognostic factors of OSCC using the Cox proportional hazard regression model 15. Statistically significant results were defined by a p value of < 0.05.

## Results

### Patient characteristics and UNC13C expression in HCC

Demographic and clinicopathological characteristics of the HCC patients are summarized in Table 1. Specimens from 265 patients were included in the current study (198 male and 67 female patients). Among the cancer patients, 25%, 100 % and 26.8% were found to have stage III/IV disease, lymph node metastasis of N0/N1 and AJCC tumour stage (III/IV), respectively, and 89.5% were moderate or poor histological grade.

**Figure 1:**
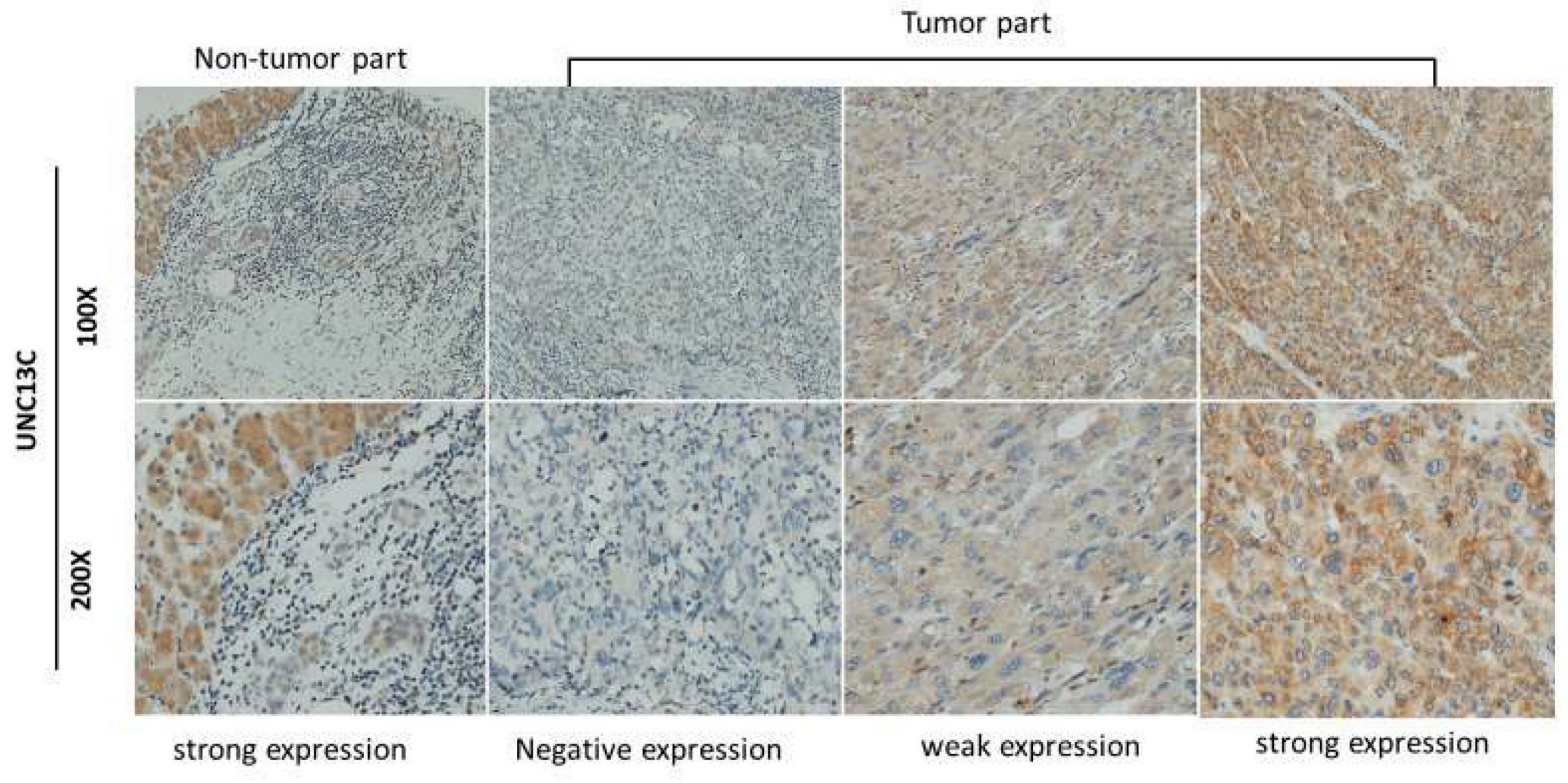
UNC13C protein expression in HCC tissues by immunohistological staining. Magnification: (top panel) 100x and lower panel (200x). Scale bars=25 and 50 µm.

The expression of UNC13C in HCC cancer tissue was examined by IHC staining. As shown in Figure 1, samples were divided into two groups based on UNC13C protein expression, as follows: 1 Low cytoplasmic UNC13C (negative expression and weak positive); 2 high cytoplasmic UNC13C (strong positive expression). The tissue samples were stratified as follows: Low UNC13C expression (n=155; 59%) and high UNC13C expression (n=110; 42%).

### Correlation between UNC13C expression and clincopathological factors

To examine the correlation between UNC13C expression levels and clinical parameters of HCC, the 265 cases were grouped based on different clinical parameters. High UNC13C expression was significantly linked to advance T stage (*p* = 0.001), advance clinical stage (p=0.001) and poor survival (*p =* 0.018) of human patients with HCC (Table 2). No significant correlation was observed in HCC patients between UNC13C expression and age, histological grade, lymph node metastasis, distant metastasis, smoking and drinking.

### Independent risk factors affect HCC outcomes

To investigate the independent prognostic value of UNC13C expression levels, univariate and multivariate Cox regression analyses were performed (Table 3). Potential prognostic factors, including UNC13C expression level, gender, drinking, pathohistological pattern, clinical stage, T classification, N classification were analysed by using the Cox proportional hazard regression model. Univariate and multivariate analyses both revealed that the overall survival rate of HCC patients was significantly linked to the expression of UNC13C (p=0.004, 95% CI 1.146-2.037; p=0.042 95% CI 1.011-1.822, respectively), T status (p<0.001, 95% CI 2.240-4.189), lymph node metastasis (p<0.001, 95% CI 2.826-11.237; p=0.001, 95% CI 1.818-10.064, respectively), distance metastasis (p=0.018, 95% CI 1.208-7.255), stage (p<0.001, 95% CI 2.360-4.369 and drinking (p=0.015, 95% CI, 1.082-2.070; p=0.005, CI 1.158-2.252, respectively).

**Figure 2:**
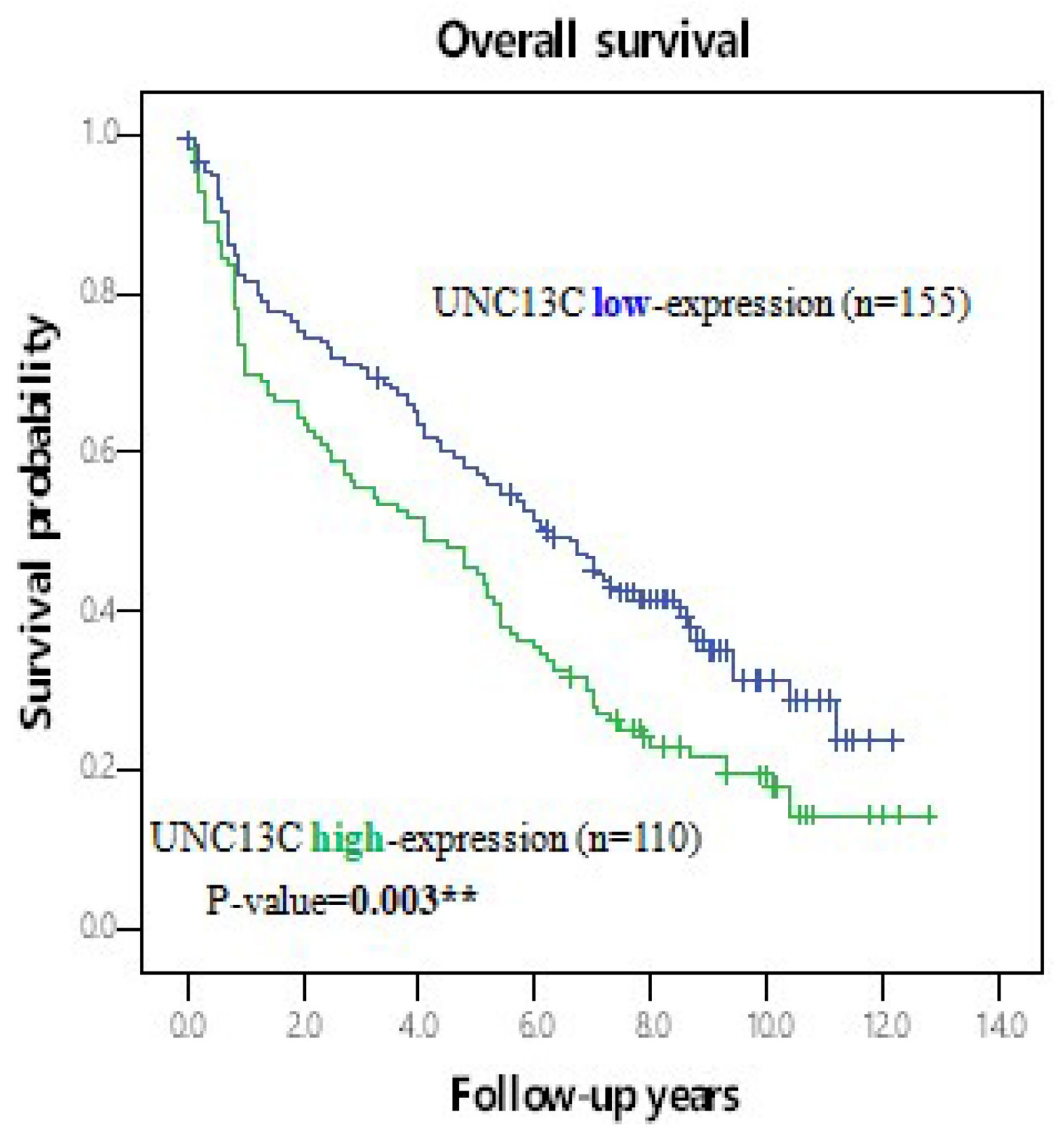
Kaplan-Meier survival analysis of UNC13C protein expression in HCC patients by log-rank tests. *p < 0.05.

Furthermore, the potential prognoses of UNC13C for OS in HCC patients was evaluated by comparing the survival time survival time of patients with high UNC13C expression to patients with low UNC13C expression. Kaplan-Meier cumulative survival curves with a log-rank test showed that long-term survival rate was significantly lower in patients with high UNC13C expression (*p* = 0.003; Figure 2). Collectively, these results demonstrated that UNC13C was an independent prognostic factor in HCC patients.

## Discussion

HCC is a global disease; that often occurs in the setting of chronic liver disease and cirrhosis. More than 60% of HCC are diagnosed in advanced stages late 16 thus causes low overall five-year survival rate (<16%) 17. Therefore to increase survival and to improve the patient´s quality life identification of molecular biomarkers and adequate therapy are crucial 18. In the current study, we analysed UNC13C protein expression levels within the perspective of the prognosis of HCC patients.

In the current study, we found lower UNC13C expression in HCC patients (Figure 1), which is not consistent with our previous findings in OSCC 10, 19. We also determined that high UNC13C expression is related to poor overall survival outcomes in HCC patients (Figure 2) suggests that it could perhaps be used as a prognostic indicator for use in HCC risk classification.

Further, we used HCC clinical tissue samples to characterize the correlation between UNC13C expression and clinicopathologic factors. High cytoplasmic UNC13C expression was strongly linked to AJCC stage, T stage, and survival (Table 2). Univariate and multivariate analyses both identified UNC13C expression, histological grade, T status, lymph node metastasis, distant metastasis, disease stage and drinking as key independent prognostic factors impacting the overall survival of HCC patients. Meta-analysis suggested that alcohol consumption is one of the possible risk factors for Intrahepaticcholangiocarcinoma (ICC) 20. To the best of our knowledge, this is the first study to evaluate the expression pattern of UNC13C and susceptibility to HCC in association with alcohol drinking status. The interaction between alcohol consumption and UNC13C expression remains unsolved. Our results indicate that UNC13C may have tumour oncogene function in HCC cells, which is not in line with earlier research 10. This was the first study to examine the use of clinicopathological factors and UNC13C protein expression within the perspective of HCC prognosis.

In conclusion, our findings demonstrate that UNC13C protein is aberrantly expressed in HCC tissues, and that protein expression levels were associated with T status, lymph node metastasis, distant metastasis, disease stage, drinking and survival. Our results from 265 HCC patients show a strong link between UNC13C expression level and survival rates. UNC13C overexpression in tumours could be a potential biomarker for prognosis and a potential target for therapy. In order to explore the potential functions and mechanisms of UNC13C and its relationship with therapeutic drugs, it is necessary to further analyse the function of UNC13C in HCC based including cell culture studies and animal experiments.

## Figures and Tables

**Table 1:** Demographic and characteristics of HCC patients.

Factors	Total Number (n=265)	%
Gender		
Male	198	74.7
Female	67	25.3
Age (Year)		
Range	16-103	
Mean	61.5	
Medium	64.0	
T (Tumor size)		
I	123	46.4
II	76	28.7
III	44	16.6
IV	22	8.3
N (Lymph node)		
N0	255	96.2
N1	10	3.8
M (Metastasis)		
M0	259	97.7
M1	6	2.3
AICC cancer stage		
I	121	45.7
II	73	27.5
III	65	24.5
IV	6	2.3
Histological grade		
Well	28	10.6
Moderate	151	57.0
Poor	86	32.5

**Table 2:** Clinicopathologic factors associated with UNC13C expression in HCC.

Cytoplasmic Staining of UNC13C				
Variable	Low	High	(n =265)	p-Value
Age	62.36±13.0	60.35±14.1		0.366
Gender				
Male	110	88	198	
Female	45	22	67	0.096
Histological grade				
Well	18	10	28	
Moderate	92	59	151	
Poor	45	41	86	0.349
T status				
T1, T2	129 (64.8%)	70 (35.2%)	199	
T3, T4	26 (39.4%)	40 (60.6%)	66	0.001***
Lymph Node Metastasis				
No	150	105	255	
Yes	5	5	10	0.746
Distance Metastasis				
M0	151	108	259	
M1	4	2	6	1.000
Stage				
I, II	126 (64.9%)	68 (35.1%)	194	
III, IV	29 (40.8%)	42 (59.2%)	71	0.001***
Smoking				
No	116	72	188	
Yes	39	38	77	0.097
Drinking				
No	118	85	203	
Yes	37	25	62	0.828
Survival				
≤3 year	47 (49.0%)	49 (51.0%)	96	
>3 year	108 (63.9%)	61 (36.1%)	169	0.018*

**Table 3:** Overall survival of UNC13C and clinicopathologic variables of patients with HCC.

		Univariate			Multivariate	
Variable	HR	95% CI	P-Value	HR	95% CI	P-Value
Expression of UNC13C						
Low	1.0			1.0		
High	1.5	1.146-2.037	0.004**	1.4	1.011-1.822	0.042*
T status						
T1, T2	1.0			1.0		
T3, T4	3.1	2.240-4.189	<0.001***	2.2	0.683-7.392	0.183
Lymph Node Metastasis						
No	1.0			1.0		
Yes	5.6	2.826-11.237	<0.001***	4.3	1.818-10.064	0.001***
Distance Metastasis						
M0	1.0			1.0		
M1	3.0	1.208-7.255	0.018*	1.4	0.516-4.055	0.483
Stage						
I, II	1.0			1.0		
III, IV	3.2	2.360-4.369	<0.001***	1.3	0.378-4.424	0.681
Drinking						
No	1.0			1.0		
Yes	1.5	1.082-2.070	0.015*	1.6	1.158-2.252	0.005**

HR (Hazard ratio) was adjusted for gender and age; * p < 0.05; ** p < 0.01; *** p < 0.001.
